# Supplementary material for: Multi-Omics Revealed Peanut Root Metabolism Regulated by Exogenous Calcium under Salt Stress
Source: Plants (Basel). 2023 Aug 31;12(17):3130. doi: 10.3390/plants12173130 (PMC10490012; doi:10.3390/plants12173130)
Supplement: Supplementary file 1 [file plants-12-03130-s001.zip › Supplementary figure S2.pdf]

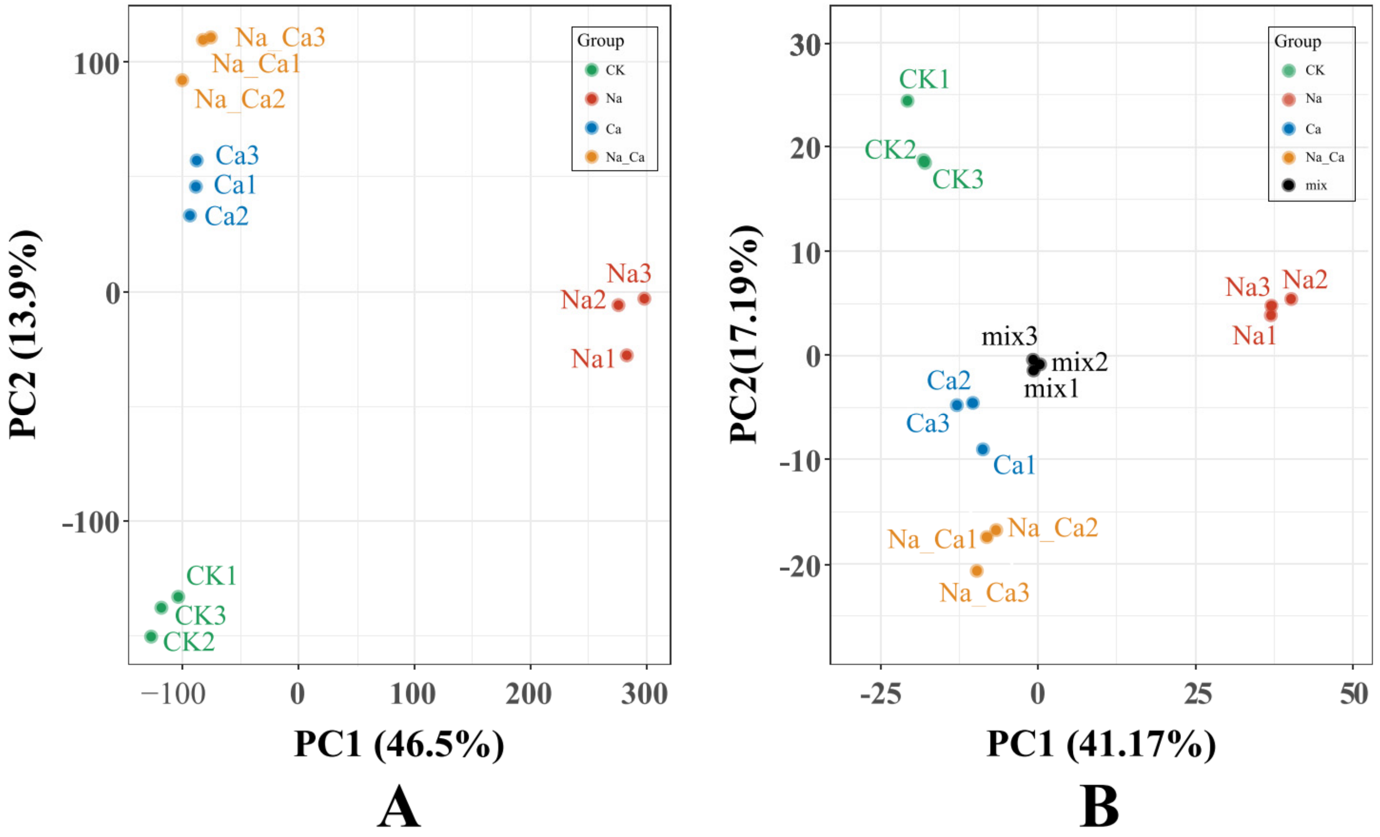

Figure S2 Two-dimensional projection scatter plot of principal components of transcriptome and metabolomics test samples.

Figure S2-A: Two-dimensional projected scatter diagram of principal components of transcriptome sequencing samples.

Figure S2-B: Two-dimensional projected scatter diagram of principal components of broadly targeted metabolomics sequencing samples.

- *Treatments: CK, untreated; Na, treated with 150 mmol/L NaCl; Ca, treated with 15 mmol/L $CaCl_2$; Na_Ca, 150 mmol/L NaCl and 15 mmol/L $CaCl_2$ Co-treatment; Mix, mixed samples of 4 treats above for metabonomic testing quality control.*
- *PC1, the first principal component; PC2, the second principal component*
