# Supplementary material for: Multi-Omics Revealed Peanut Root Metabolism Regulated by Exogenous Calcium under Salt Stress
Source: Plants (Basel). 2023 Aug 31;12(17):3130. doi: 10.3390/plants12173130 (PMC10490012; doi:10.3390/plants12173130)
Supplement: Supplementary file 1 [file plants-12-03130-s001.zip › Supplementary figure S3.pdf]

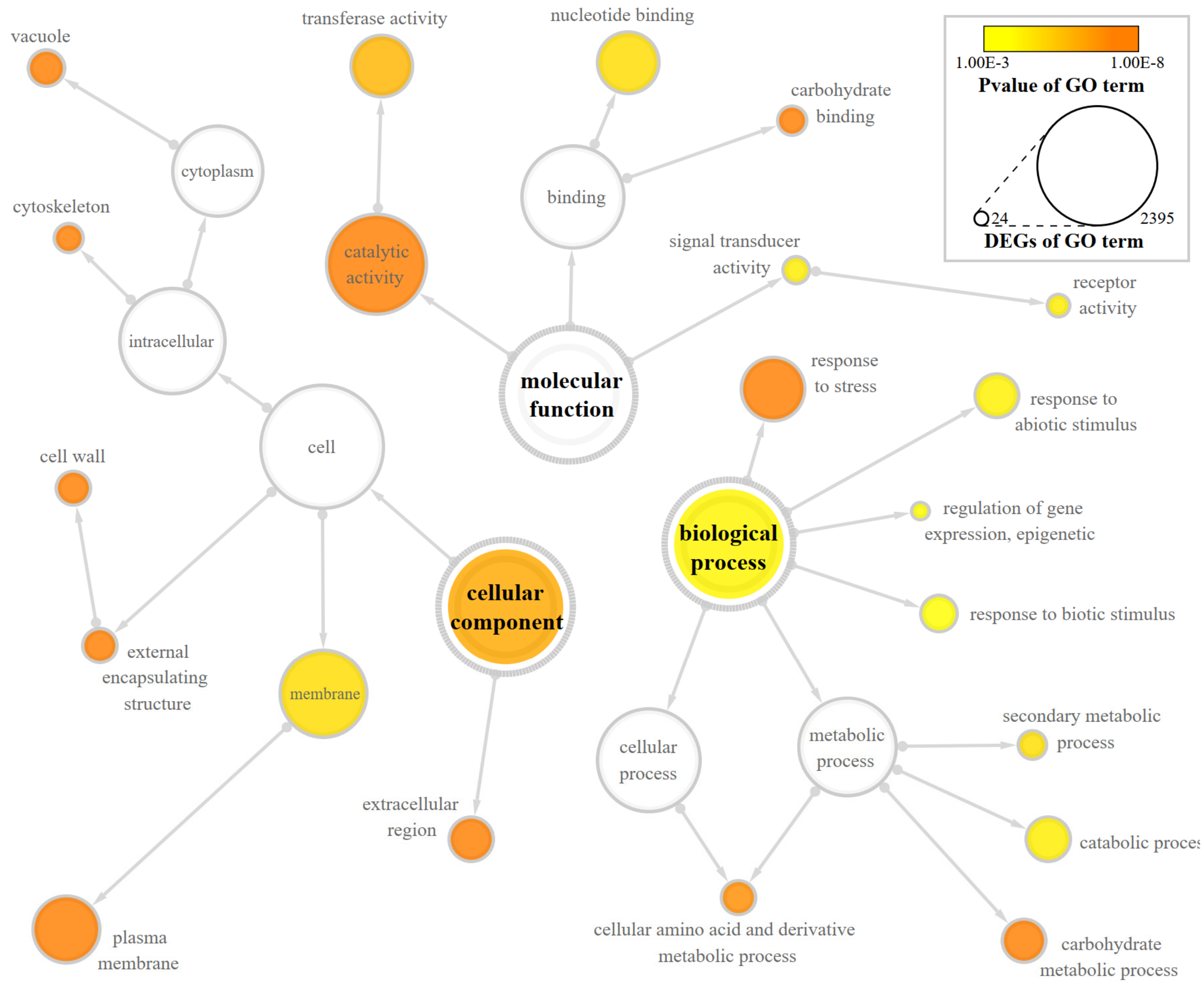

Figure S3 GO slim analysis of Differently expressed genes (DEGs) shared by 2 pairwise comparison groups.

- *Treatments: CK, untreated; Na, treated with 150 mmol/L NaCl; Ca, treated with 15 mmol/L $CaCl_2$; Na_Ca, 150 mmol/L NaCl and 15 mmol/L $CaCl_2$ Co-treatment.*
- *2 pairwise comparison groups: Na v.s. CK and Na_Ca v.s. Na (experimental group v.s. control group).*
- *GO enrichment analysis is calculated and visualized by BiNGO plug-in of catoscape software.*
