# Supplementary material for: Multi-Omics Revealed Peanut Root Metabolism Regulated by Exogenous Calcium under Salt Stress
Source: Plants (Basel). 2023 Aug 31;12(17):3130. doi: 10.3390/plants12173130 (PMC10490012; doi:10.3390/plants12173130)
Supplement: Supplementary file 1 [file plants-12-03130-s001.zip › Supplementary figure S4.pdf]

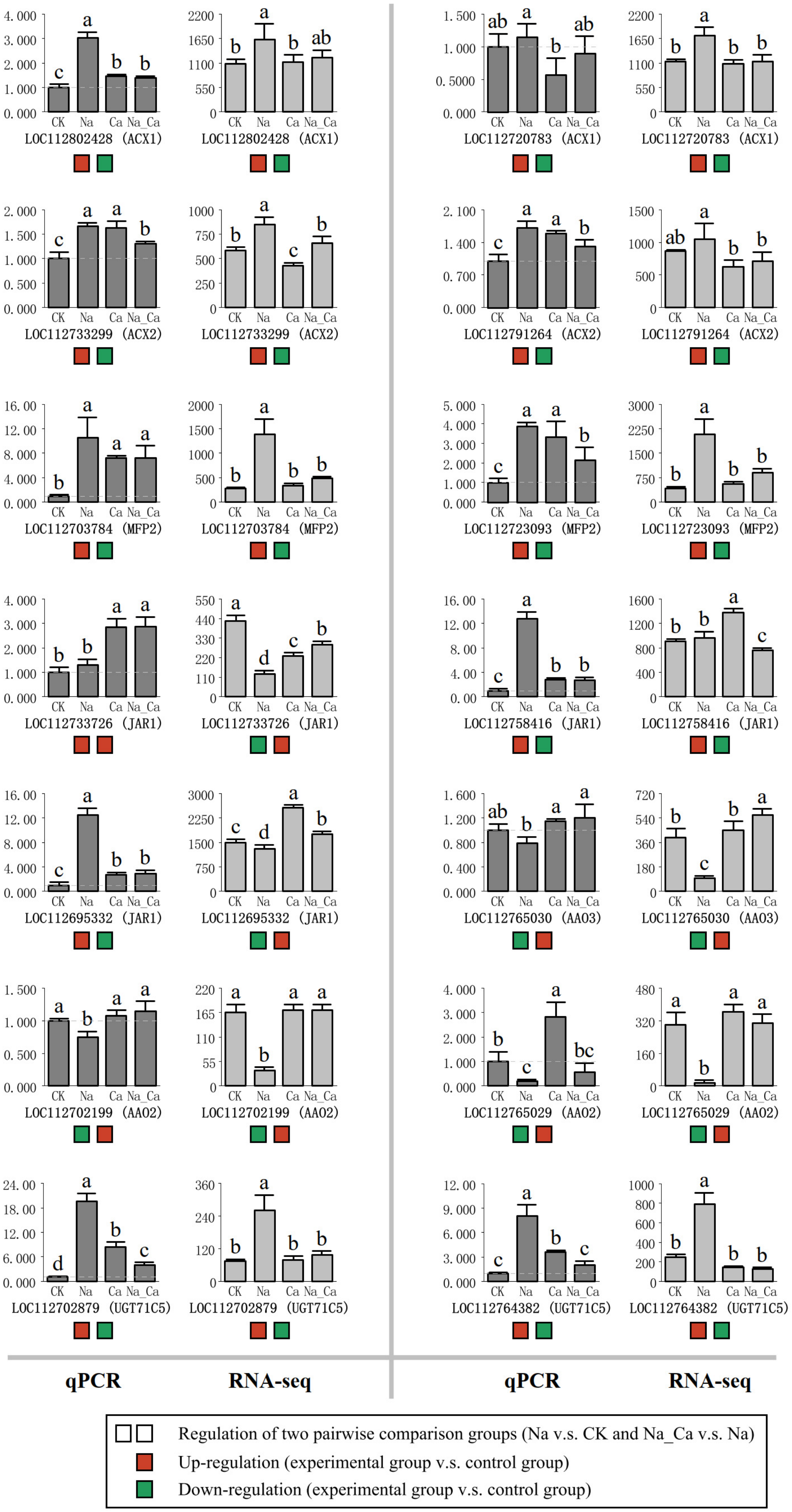
LOC112802428 (ACX1)
LOC112802428 (ACX1)
LOC112720783 (ACX1)
LOC112720783 (ACX1)
LOC112733299 (ACX2)
LOC112733299 (ACX2)
LOC112791264 (ACX2)
LOC112791264 (ACX2)
LOC112703784 (MFP2)
LOC112703784 (MFP2)
LOC112723093 (MFP2)
LOC112723093 (MFP2)
LOC112733726 (JAR1)
LOC112733726 (JAR1)
LOC112758416 (JAR1)
LOC112758416 (JAR1)
LOC112695332 (JAR1)
LOC112695332 (JAR1)
LOC112765030 (AAO3)
LOC112765030 (AAO3)
LOC112702199 (AAO2)
LOC112702199 (AAO2)
LOC112765029 (AAO2)
LOC112765029 (AAO2)
LOC112702879 (UGT71C5)
LOC112702879 (UGT71C5)
LOC112764382 (UGT71C5)
LOC112764382 (UGT71C5)
CK Na Ca Na_Ca
qPCR
RNA-seq
qPCR
RNA-seq
Regulation of two pairwise comparison groups (Na v.s. CK and Na_Ca v.s. Na)
Up-regulation (experimental group v.s. control group)
Down-regulation (experimental group v.s. control group)

Figure S4 Comparison of expression trend between 2 pairwise comparison groups of 14 differentially expressed genes (DEGs) under two test methods (qPCR and RNA-seq).

- *Treatments: CK, untreated; Na, treated with 150 mmol/L NaCl; Ca, treated with 15 mmol/L $CaCl_2$; Na_Ca, 150 mmol/L NaCl and 15 mmol/L $CaCl_2$ Co-treatment.*
- *2 pairwise comparison groups: Na v.s. CK and Na_Ca v.s. Na (experimental group v.s. control group).*
