# Supplementary figures and images for: Multi-Omics Revealed Peanut Root Metabolism Regulated by Exogenous Calcium under Salt Stress
Source: Plants (Basel). 2023 Aug 31;12(17):3130. doi: 10.3390/plants12173130 (PMC10490012; doi:10.3390/plants12173130)

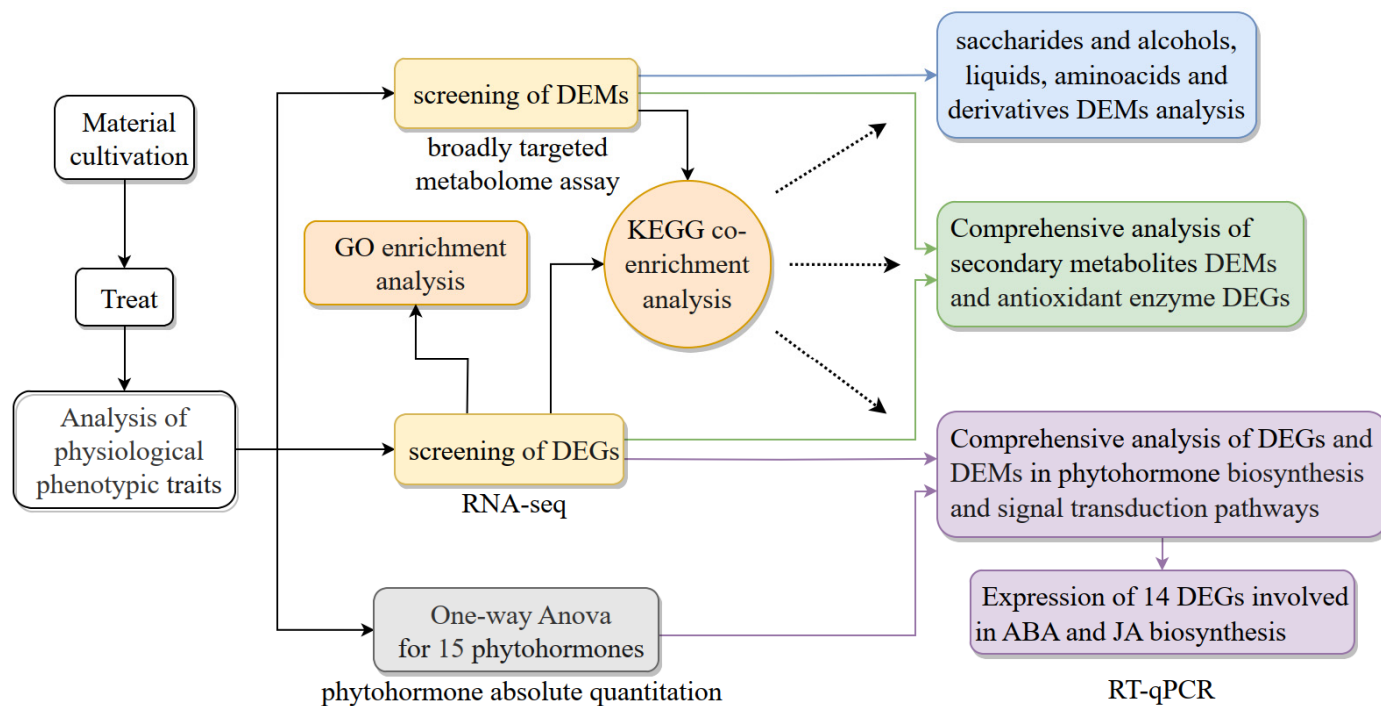

**Figure S1** An overview of the experimental data analysis workflow.

Supplement: Supplementary file 1 [file plants-12-03130-s001.zip › Supplementary figure S1.pdf]
